# Pamidronic acid and cabergoline as effective long-term therapy in a 12-year-old girl with extended facial polyostotic fibrous dysplasia, prolactinoma and acromegaly in McCune-Albright syndrome: a case report

**DOI:** 10.1186/1752-1947-6-32

**Published:** 2012-01-24

**Authors:** Carl Friedrich Classen, Monika Mix, Ulrike Kyank, Christina Hauenstein, Dieter Haffner

**Affiliations:** 1 University Children's Hospital, Ernst-Heydemann-Strasse 8, D-18057 Rostock, Germany; 2 Institute for Radiology, Rostock, Ernst-Heydemann-Strasse 8, D-18057 Rostock, Germany; 3 Department of Pediatric Kidney-, Liver- and Metabolic Diseases, Hannover Medical School, Carl-Neuberg Str. 1, 30625 Hannover, Germany

## Abstract

**Introduction:**

McCune-Albright syndrome is a complex inborn disorder due to early embryonal postzygotic somatic activating mutations in the *GNAS*1 gene. The phenotype is very heterogeneous and includes polyostotic fibrous dysplasia, typically involving the facial skull, numerous café-au-lait spots and autonomous hyperfunctions of several endocrine systems, leading to hyperthyroidism, hypercortisolism, precocious puberty and acromegaly.

**Case presentation:**

Here, we describe a 12-year-old Caucasian girl with severe facial involvement of fibrous dysplasia, along with massive acromegaly due to growth hormone excess and precocious puberty, with a prolactinoma. Our patient was treated with a bisphosphonate and the prolactin antagonist, cabergoline, resulting in the inhibition of fibrous dysplasia and involution of both the prolactinoma and growth hormone excess. During a follow-up of more than two years, no severe side effects were noted.

**Conclusion:**

Treatment with bisphosphonates in combination with cabergoline is a suitable option in patients with McCune-Albright syndrome, especially in order to circumvent surgical interventions in patients suffering from polyostotic fibrous dysplasia involving the skull base.

## Introduction

McCune-Albright syndrome is a complex inborn disorder due to early embryonal postzygotic somatic activating mutations in the *GNAS1 *gene, which codes for a number of different transcripts by alternative promoters and alternative splicing. Among these, the Gs-alpha, the XLAS, the NESP55 and the A/B transcripts are involved in the regulation of bone metabolism and endocrine functions [1-4].

The full phenotype of McCune-Albright syndrome includes fibrous dysplasia, café-au-lait spots and autonomous hyperfunctions of several endocrine pathways [2,5,6]. Bone disease may present as polyostotic or monostotic fibrous dysplasia. Polyostotic involvement-characterized by multiple separated foci of dysplasia-is typically seen in the face but also in the extremities, or both. Fibrous dysplasia of the extremities predisposes for pathological fractures. Massive thickening of the facial bones may lead to bizarre disfigurement with cosmetic and psychosocial sequelae. Further, the encasement of cranial nerves carries the risk of function loss, like visual impairment; however, as recently shown by a larger study, this is rare even in extended cases [7]. Sometimes the syndrome may lead to enhanced intracranial pressure.

Autonomous hyperfunction in the endocrine glands may typically manifest as precocious puberty or acromegaly, but also primary hyperthyroidism or hypercortisolism. An excess of growth hormone and prolactin is, in most cases, due to the diffuse hyperfunction of the anterior pituitary lobe, but sometimes adenomas are found [1]. A growth hormone excess and lesions of fibrous dysplasia have mutual effects on phosphate levels, as growth hormone-mediated phosphate reabsorption is counteracted by phosphaturic substances secreted from fibrous dysplastic lesions. Thus, acromegaly in McCune-Albright syndrome has several features that are specific to this disease [1]. In rare cases, hepatobiliary disorders, cardiomyopathy, proximal tubulopathies, nephrocalcinosis and hypophosphatemic rickets are observed in McCune-Albright syndrome [1-4].

The syndrome is very polymorphic and the degree of skeletal or endocrine abnormality is heterogeneous. Thus it should be considered in all cases of fibrous dysplasia or in pituitary hyperfunctions [1,6].

Here, we describe a case of McCune-Albright syndrome with severe facial disfigurement due to fibrous dysplasia, acromegaly, precocious puberty and a pituitary adenoma. The girl was successfully treated over years with pamidronic acid to inhibit progressive fibrosis and cabergoline as a prolactin antagonist effective against the adenoma.

### Case presentation

Our patient was the third child of non-consanguineous Caucasian parents; her father died from cystic kidney disease when she was three years old. Her birth and neonatal development were uneventful.

**Figure 1:**
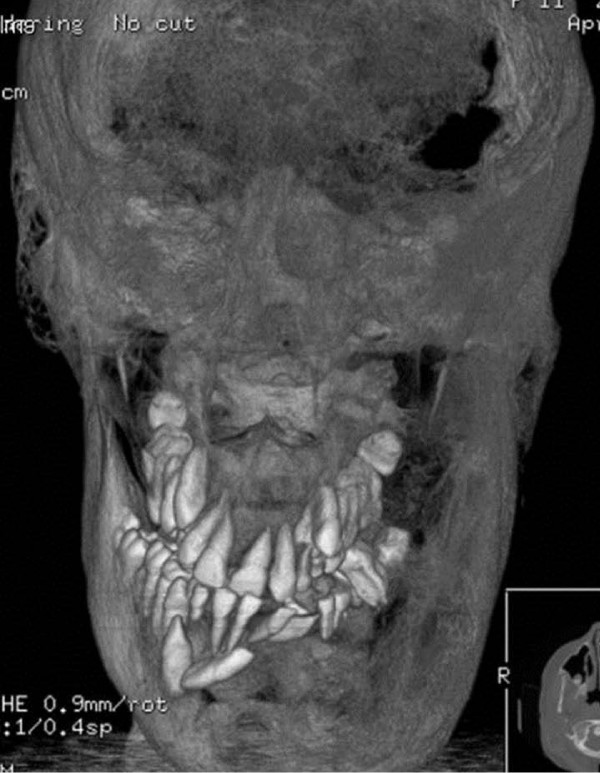
**Three-dimensional reconstruction with volume rendering from a computed tomography scan of her skull**. Shows facial disfigurement due to extended bone distensions with mixed radiolucent and sclerotic focal lesions.

At the age of three years, skull asymmetry became visible, which was more and more pronounced in the following years. Fibrous dysplasia was identified by computed tomography, involving in particular the left skull base with the optic canal. Café-au-lait spots were not visible. Beginning at the age of five, the girl underwent five subsequent surgical interventions for plastic reconstruction of her face. However, after each surgery, the girl experienced a marked regrowth of the dysplasia leading to massive facial disfigurement (Figure 1).

**Figure 2:**
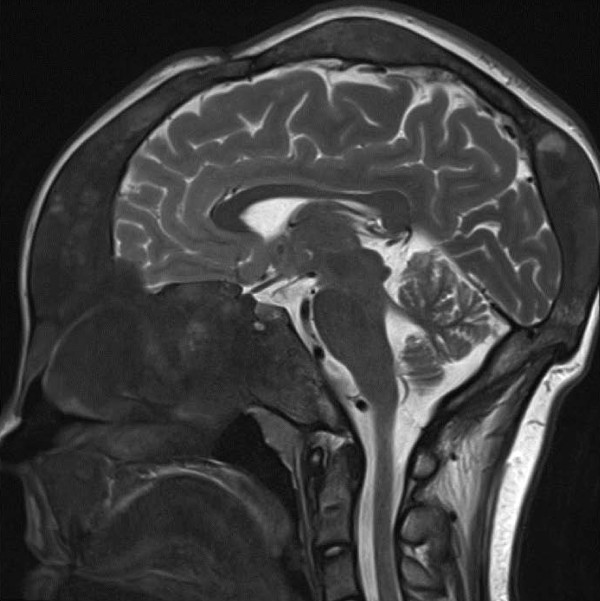
**T2-weighed sagittal magnetic resonance image**. Shows massive thickening of her skull base due to fibrous dysplasia and a pituitary adenoma.

Finally, at the age of eleven years, our patient presented with gigantism, leading to endocrinological examinations. Her pubertal development now showed Tanner stage PH2-3, B2-3; her body weight was 80.8 kg and length 175.2 cm (both above than the 97th percentile). Besides the asymmetrical skull dysplasia, the girl also presented with an acromegalic appearance. Laboratory evaluations revealed a massive hyperprolactinemia of 5475 mU/L (normal range < 500 mU/L), high growth hormone levels (that is, 43.2 mU/mL at 8 a.m.), and an elevated insulin-like growth factor 1 (IGF-1) of 892 ng/mL (age-related normal range, 111 ng/mL to 693 ng/mL). Her alkaline phosphatase was 690 U/L (normal range, 51 U/L to 332 U/L). A pituitary adenoma was seen on magnetic resonance imaging, located within the massively expanded dysplastic skull base (Figure 2). An ophthalmological examination revealed neither diplopia nor any other subjective impairment of her vision. There was a mild paleness of her optic nerve papilla and mild left-sided hemianopia.

The case was discussed in a multidisciplinary conference for the four problems, that is, her facial disfigurement, possible danger to her left visual nerve and the pituitary tumor leading to local pressure and acromegaly. Besides the futility and probable enormous difficulty of cosmetic surgery, our patient and her mother refused any further surgical interventions for this purpose. A surgical intervention at her pituitary gland appeared difficult due to its complete impaction within the dysmorphic, fibrous bone of her skull base. Therefore, we did not obtain a histopathology specimen, and so it was not possible to tell whether the pituitary tumor was a lesion of acidophils, prolactin cells or both. Radiotherapy of her pituitary gland by conventional or gamma-knife irradiation was considered as too toxic, especially since malignant transformation is a well-known possible late effect, particularly in fibrous dysplasia tissues [8].

Thus, based on published experiences in McCune-Albright syndrome [1,2,9,10], at the age of twelve years, our patient started pharmacological treatment. First, to treat the fibrous dysplasia, we initiated parenteral bisphosphonate therapy with pamidronic acid (Aredia), given every four weeks with a dosage of 30 mg, which equals about 0.4 mg per kilogram body weight. This was done by continuous infusion over four hours with cardiorespiratory monitoring and included regular examination of her kidney and liver function parameters.

In parallel, cabergoline, as the current standard therapy of prolactinomas, was given with a stepwise escalation of the dosage to reach a level of 2 mg twice weekly, which was tolerated well. Regular controls of blood pressure and cardiac function were included.

The clinical course was monitored regularly in monthly to three monthly intervals. The therapy was tolerated well. No acute side effects, such as rash, nausea, electrolyte disturbances, cardiovascular, pulmonary or renal impairment, occurred. Further, the continuous treatment, which has been extended without change for two and a half years, was not associated with osseous necrosis of the jaw or elsewhere, nephrocalcinosis or cardiac valve fibrosis.

Magnetic resonance imaging showed no further progression of the fibrous dysplasia; her osseous status has remained unchanged for two and a half years. Her alkaline phosphatase, initially elevated up to 690 U/L, declined to 587 U/L after one year, and 417 U/L after two years of therapy.

Her visually evoked potentials showed a mild prolongation after the first six months of therapy, which did not go along with any subjective symptoms.

Endocrinological tests showed an immediate and rapid response of prolactin, which fell to the normal range within two months of therapy and have remained completely suppressed since. In parallel, we observed a normalization of her growth hormone levels, and her IGF-1 level fell to normal levels after four months. The growth of our patient slowed down, her final length of 180 cm was, however, much higher than her mid parental expectation. Our patient achieved menarche about one year after the initiation of treatment, that is, several months after normalization of her prolactin levels. Magnetic resonance imaging of her skull base showed a marked shrinkage of the hypophyseal tumor.

In summary, we observed stabilization both of the osseous and the endocrinological symptoms of the disease in our patient, allowing a significant improvement in the quality of life of our patient without further surgical interventions.

## Discussion

The clinical symptoms of McCune-Albright syndrome in our patient were related to both bone and endocrine involvement, that is, facial disfigurement caused by excessive bone growth and acromegaly with visual nerve encasement, and the growth of a pituitary tumor. For both problems, surgical treatment was not applicable.

It has recently been described that treatment with bisphosphonates may be an effective measure in fibrous dysplasia [1,5,6,10], although randomized trials are lacking. Chemically, bisphosphonates, among which pamidronic acid is commonly used, are simple pyrophosphate derivatives interfering with phosphate metabolism in osteoclasts. This leads to inhibition or, ultimately, apoptosis of these cells [10]. Typically, bisphosphonates are used in the treatment of osteolytic metastases of adult carcinomas, both in order to reduce pain and to slow tumor growth or progression [11].

In children and adolescents, bisphosphonate therapy appears to be well tolerated in general, even in long-term treatment [10,12]. In these cases, they are applied in rare disorders of calcification, like osteogenesis imperfecta, congenital or malignoma-induced hypercalcemia, osteoporosis, or, as in our case, fibrous dysplasia.

Side effects of bisphosphonates include acute phase reactions, dyspepsia, esophagitis, iritis or osteonecrosis, especially in the jaw. However, in general, the treatment is well tolerated in children and adolescents; particularly, osteonecrosis is practically absent [5,10]. Several authors have already described a partial or complete arrest of fibrous dysplasia on bisphosphonate treatment in children and adolescents [2], with good tolerance even upon long-term treatment. Chan and Zacharin observed a progress of fibrous dysplastic lesions in spite of pamidronic acid treatment in long bones, but an arrest of fibrous dysplasia in facial bones [13].

Cabergoline is a synthetic ergot alkaloid which performs as a long acting D2-selective dopamine agonist, inhibiting D2 receptor-mediated prolactin secretion in the anterior lobe of the pituitary gland [14]. Pharmacological treatment of pituitary adenomas by cabergoline primarily aims at inhibition of prolactin stimulation, leading to the involution of prolactinomas. In recent years, it been shown that cabergoline is more effective than the previous standard, bromocriptine. However, some efficacy has also been described in growth hormone inhibition and inhibition of other anterior pituitary hormones, such as adrenocorticotropic hormone [1]. In a larger cohort, Akintoye *et al. *observed normalization of IGF-1 in most patients treated with cabergoline, long-acting octreotide, or both [1].

Side effects of cabergoline include arterial hypotonia, fatigue, depression, dyspepsia, nausea and vomiting, cramps and erythema; an effect of particular concern is cardiac valve pathology, especially valve thickening [14]. The relevance of this has recently been questioned [15]. In our patient, we observed no relevant side effects; in particular, we saw no cardiac abnormalities after more than two years of continuous treatment.

The combination of prolactinoma with growth hormone excess is typical in McCune-Albright syndrome, leading to an early manifestation acromegaly that may enhance the asymmetric facial disfigurement [5,14]. In cases in which cabergoline alone is not sufficient to stop growth hormone excess, the somatostatin analogue octreotide or the growth hormone receptor antagonist pegvisomant may be additionally beneficial for the treatment of acromegaly [3,11]. In our patient, this appeared not to be necessary, since her IGF-1 levels went down to normal and the development of acromegaly was arrested.

## Conclusion

Thus, treatment with bisphosphonates in combination with cabergoline successfully arrested both her dysplastic bone growth and endocrine malfunction, without severe side effects. We believe that this approach is a suitable option in patients with McCune-Albright syndrome, especially in order to circumvent surgical interventions that might be of particular risk in patients suffering from polyostotic fibrous dysplasia involving the skull base.

## Consent

Written informed consent was obtained from the mother (legal guardian) of the patient for publication of this case report and any accompanying images. A copy of the written consent is available for review by the Editor-in-Chief of this journal.

## Competing interests

The authors declare that they have no competing interests.

## Authors' contributions

CFC performed the basic organization of the writing and clinical data collection and drafted the manuscript. MM carried out the endocrinological consulting and analyzed and interpreted the patient data regarding the clinical presentation. UK carried out the clinical consulting and analyzed the patient data. CH selected the imaging and participated in the design and coordination of the work and helped to draft the manuscript. DH added particular background information and was a major contributor in writing the manuscript. All authors read and approved the final manuscript.
